# Pathogenic Fungal Infection in the Lung

**DOI:** 10.3389/fimmu.2019.01524

**Published:** 2019-07-03

**Authors:** Zhi Li, Gen Lu, Guangxun Meng

**Affiliations:** ^1^The Joint Center for Infection and Immunity, Guangzhou Women and Children's Medical Center, Guangzhou Institute of Pediatrics, Guangzhou, China; ^2^The Joint Center for Infection and Immunity, Institute Pasteur of Shanghai, Chinese Academy of Science, Shanghai, China

**Keywords:** pulmonary fungal infection, pattern recognition receptor, inflammasome, cytokine, chemokine

## Abstract

Respiratory fungal infection is a severe clinical problem, especially in patients with compromised immune functions. *Aspergillus, Cryptococcus, Pneumocystis*, and endemic fungi are major pulmonary fungal pathogens that are able to result in life-threatening invasive diseases. Growing data being reported have indicated that multiple cells and molecules orchestrate the host's response to a fungal infection in the lung. Upon fungal challenge, innate myeloid cells including macrophages, dendritic cells (DC), and recruited neutrophils establish the first line of defense through the phagocytosis and secretion of cytokines. Natural killer cells control the fungal expansion in the lung via the direct and indirect killing of invading organisms. Adaptive immune cells including Th1 and Th17 cells confer anti-fungal activity by producing their signature cytokines, interferon-γ, and IL-17. In addition, lung epithelial cells (LEC) also participate in the resistance against fungal infection by internalization, inflammatory cytokine production, or antimicrobial peptide secretion. In the host cells mentioned above, various molecules with distinct functions modulate the immune defense signaling: Pattern recognition receptors (PRRs) such as dectin-1 expressed on the cell surface are involved in fungal recognition; adaptor proteins such as MyD88 and TRAF6 are required for transduction of signals to the nucleus for transcriptional regulation; inflammasomes also play crucial roles in the host's defense against a fungal infection in the lung. Furthermore, transcriptional factors modulate the transcriptions of a series of genes, especially those encoding cytokines and chemokines, which are predominant regulators in the infectious microenvironment, mediating the cellular and molecular immune responses against a fungal infection in the lung.

## Introduction

With the increasing number of immunocompromised patients, diseases caused by fungal infections remain a great threat in public health. Opportunistic fungi, including *Aspergillus* with invasive aspergillosis (1–3), *Cryptococcus* with cryptococcosis (4–6), *Pneumocystis* with pneumonia (7), and endemic fungi (8, 9) are the main sources of fungal infections in the lungs of humans. Although these infections are rarely found in the target organs in healthy people, they may result in life-threatening invasive diseases in patients with an impaired immune system. These individuals include patients suffering immunodeficiency disorders such as HIV/AIDS and cancer patients who undergo chemotherapy, as well as those patients who receive immunosuppressive therapy such as in bone marrow/stem cell transplantation. Pathogenic fungal infections in the lung has resulted in the incidence and infectious death of invasive mycoses especially in patients with severe defects of host immune responses (10, 11). Some fungal pathogens initiate the infection through surface proteins from pathogen-host interaction, finally leading to mycosis with multiple tissue lesions especially in immunocompromised patients as mentioned above. For example, *Cryptococcus* mainly infects the lung and invades the brain via circulation, leading to lethal cryptococcal meningitis. Cryptococcosis, including life-threatening Cryptococcal meningoencephalitis (12), afflicts about 1 million AIDS patients and causes more than 600,000 deaths worldwide annually.

**Figure 1:**
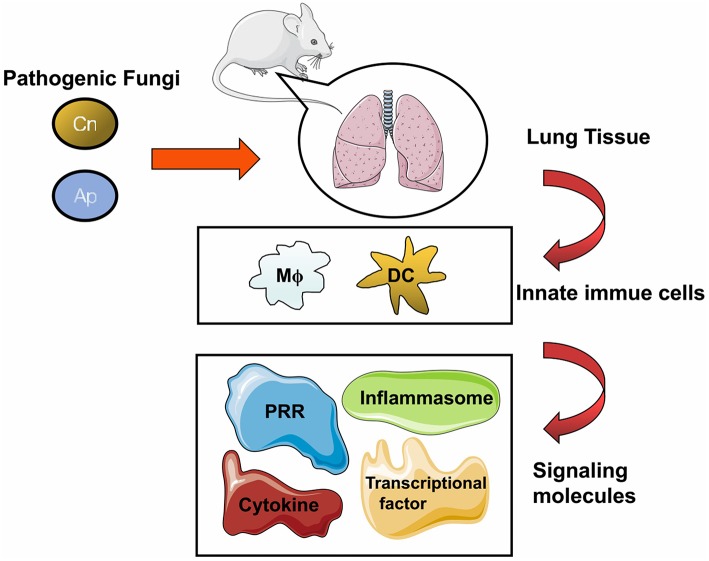
General cellular and molecular components against fungal infection in the lung. The pathogenic fungi including *Cryptococcus neoformans* (Cn), *Aspergillus* (Ap), *Pneumocystis* (Pc), and Endemic fungi (Ef) can cause pulmonary infection in human and in murine models. Fungal pathogens could trigger host immune response upon inhalation, and lung tissue is the major infectious target of these pathogens. Once the infection is initiated, multiple cells either resident in the lung tissue or recruited from blood and lymphoid organs are motivated to clear the invasive fungi. After entering host cells, molecules including receptors, adaptors, or effectors come into play with divergent roles to respond to the fungal challenge.

To minimize the damage of fungal infections, the human body has shaped a set of unique and sophisticated defense mechanisms, in which host innate immunity plays a crucial role. Aiming to eliminate the fungal dissemination, two typical innate immune cells, namely macrophage and dendritic cells (DC) as defensive troops constitute the first line in multiple organs. Furthermore, these innate cells can connect innate and adaptive immunity by serving as specific antigen-presenting cells (APCs), by which fungal antigens could be presented, to prime naïve T cells. Upon recognition of fungal pathogens, innate immune cells are activated by pathogen associated molecular patterns (PAMPs) via specific pattern recognition receptors (PRRs) on the surface, for further intracellular signaling transduction. The PRRs involved in fungal detection identified to date include Toll like receptors (TLRs), C-type lectin receptors (CLRs) as well as NOD like receptors (NLRs) (13, 14). Although breakthroughs have been made in the field of host response to mycobiota, novel cellular and molecular mechanisms on antifungal immunity remain to be established for the control of fungal infection and associated organ damage (Figure 1).

Despite antifungal drugs conferring protection against the pulmonary fungal infection (15, 16), drug resistance is still a severe problem. To reduce the incidence and death rate of pulmonary mycoses, efforts for the investigation on the mechanisms of pulmonary fungal infection, remain a requirement from both basic and clinical aspects. In the current review, we will discuss concrete research progress made on mycobiota regarding respiratory tract infections including *Aspergillus, Cryptococcus, Pneumocystis*, and endemic mycoses.

## Pathogens: The Characterization of Fungal Infections in the Lung

### *Aspergillus* and *Cryptococcus* Are the Major Fungal Pathogens in the Lung Infection

*Aspergillus* mold is one of the most common fungal species which can sufficiently sporulate with released airborne conidia. The produced conidia in the air are small enough (2 to 3 μm) to arrive at human airways and pulmonary alveoli, causing a spectrum of diseases including lethal infections in immunocompromised individuals and in atopic patients with asthma allergies (1, 17). In healthy individuals, inhaled conidia are engulfed by alveolar macrophages and killed in a phagocyte oxidase-dependent fashion (18–20). In immunocompromised individuals, incomplete killing of inhaled fungal conidia results in germination and tissue invasion by fungal hyphae (21).

Cryptococcosis is caused by *Cryptococcus* exposure to the lung after the airborne organisms' inhalation. As a subtype of *Cryptococcus, Cryptococcus neoformans* distribute widely, particularly in soil and avian habitats. The most severe outcome of *Cryptococcus* infection is cryptococcal meningitis. Because *C. neoformans* and *Cryptococcus gattii* can disseminate from the lung to invade the brain by crossing the blood brain barrier (BBB), the fungal cells directly penetrate the BBB via endothelial cells on the blood vessels of the brain, using a “Trojan horse” strategy that is involved in the transport of phagocytes (5). In animal experiments, by counting yeast cell numbers in the brain, *C. neoformans* still remained in the CNS where large scale colonization and tissue injury could occur in spite of defense mechanisms employed by the host (22).

### *Pneumocystis* and Endemic Mycoses Cause Infections in the Lung

*Pneumocystis* pneumonia (PCP) induced by fungal pathogen species *Pneumocystis* such as *Pneumocystis jirovecii* is the most common AIDS-defining disease, and is also found in non-HIV immunocompromised patients with deficiency in adaptive immunity, or individuals taking prolonged high-dose systemic glucocorticoids (11). The *Pneumocystis* antigens are primary surface glycoprotein (Msg or glycoprotein A) (23) and *Pneumocystis* protease, kexin (Kex1, Prt1). As a potential therapeutic target, Kex is supposed to participate in the proteolytic processing of *Pneumocystis* surface antigens (24, 25).

Endemic mycoses usually occur in restricted geographic areas, which can result in severe and fatal cases in hospitalizations (26, 27). In immunocompromised patients, endemic mycoses can also cause more severe and disseminated disease resulting in higher mortality (28). In many regions, increased incidence of endemic mycoses is correlated with a growing population of immunocompromised subjects (29). Surprisingly, mortality is also high in non-immunocompromised hosts (27). In North America, three major endemic mycoses including coccidioidomycosis, histoplasmosis, and blastomycosis could present as community acquired pneumonias (CAP) (27). For coccidioidomycosis in the southwest endemic areas, half of the infected people are asymptomatic, as an acute infection resembles respiratory symptoms such as pneumonia and bronchitis and a fever, which then experiences a self-limited process, and only very few cases can develop into disseminated infection; histoplasmosis, caused by infectious agent *Histoplasma. capsulatum*, are considered a community acquired infection with an exposure history to soil containing bat or bird droppings, patients also present pneumonia symptoms described as acute/chronic pulmonary histoplasmosis, severe cases may culminate in respiratory failure and death. However, blastomycosis is less common than histoplasmosis and coccidioidomycosis. As to the management of the endemic mycoses-caused community acquired pneumonia, anti-fungal treatment is deemed important, and azole therapy such as oral fluconazole is preferred (30). In addition, paracoccidioidomycosis has its geographic distribution mainly in Latin America where Brazil largely accounts for the reported cases (31).

### The Characteristics of Human Responses to Fungal Infection: A Distinct Susceptibility Between Immunocompromised and Immunocompetent Hosts

**Figure 2:**
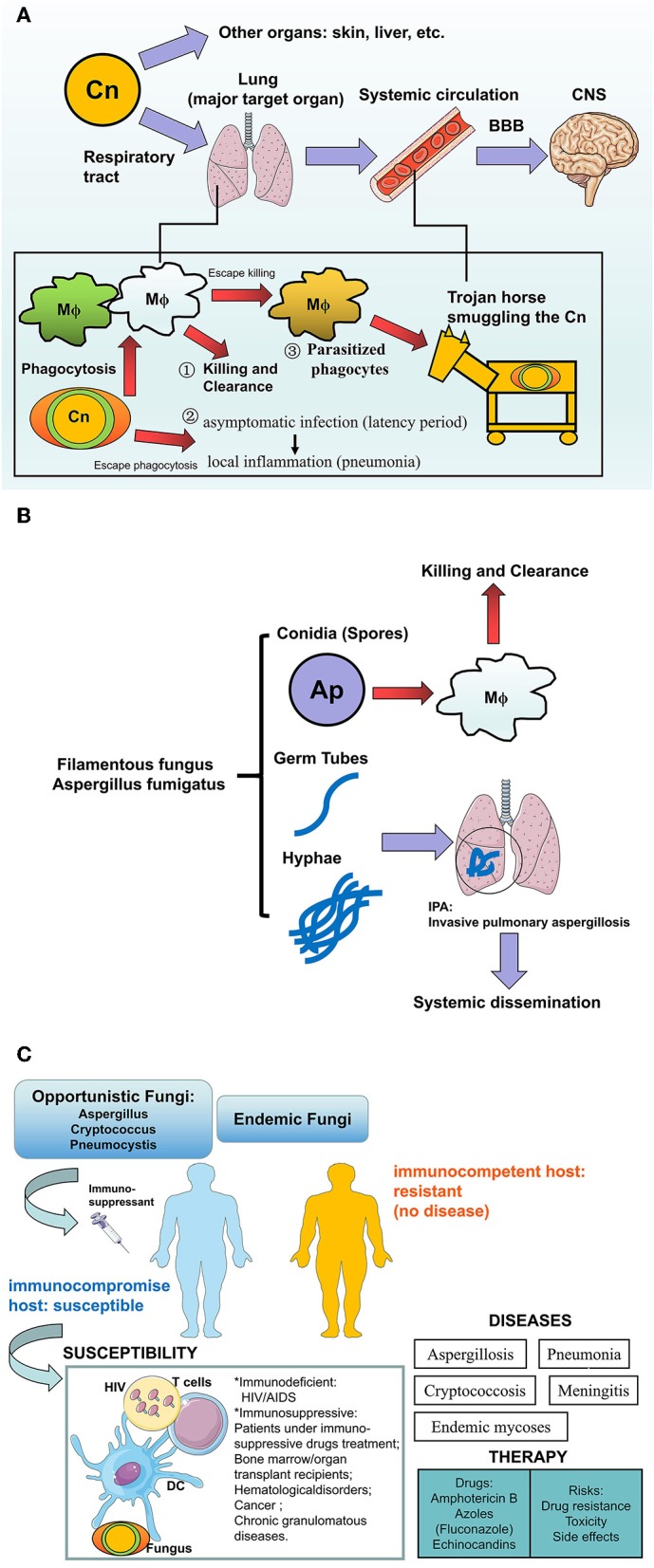
The human fungal infections in the lung. **(A)** *Cryptococcus neoformans* infection in human: how the pathogens invade into the brains? *Cryptococcus neoformans* (Cn) is more aggressive to the lung via respiratory the tract compared with other organs (skin, liver, etc.), where outcomes of fungi-host communication are: (1) Killing and clearance by macrophages after phagocytosis, (2) The encapsulated fungi are able to escape from phagocytosis and further establish virtual infection after pulmonary colonization, from asymptomatic latency period to local inflammation such as pneumonia, (3) Part of the phagocytosed fungi could escape from killing and further parasitize the host cells like a Trojan horse which transports the pathogenic fungi into the CNS via circulation. After crossing the BBB, Cn that accumulates in the CNS would result in lethal cryptococcal meningitis especially in susceptible hosts. **(B)** *Aspergillus fumigatus* in humans: Hyphae are the pathogenicity for IPA. *Aspergillus fumigatus* are a Filamentous fungus in the lung. Inhaled conidia (spores) could be ingested and killed by the alveolar macrophage constructed first line of host defense, while the escaped conidia grow into mature germ tubes and hyphae, which not only cause Invasive pulmonary aspergillosis (IPA) but also lead to systemic dissemination by invading vessels. This pathological process is more common in patients who lack an effective host defense or hyphal defense. **(C)** Summary of fungal infection to human hosts. Opportunistic fungi or endemic fungi infection usually occurs in hosts with a profound immune deficiency involving HIV/AIDS, or immunosuppressed patients involving hematopoietic stem cells (HSCs) or solid organ transplantation, immunosuppressant, or glucocorticosteroid therapy, hematological disorders (leukemia, lymphoma), cancer, and chronic granulomatous diseases. In susceptible hosts, multiple factors participated in this process including HIV, Fungus, immune cells, or even the immunosuppressant and transplanted organs. Infection and subsequent inflammation result in diseases e.g., Mycoses, pneumonia, and meningitis. The current anti-fungal treatments include azoles and Amphotericin B, but potential risks also lead to the ineffectiveness of the drugs.

The clinical case of human cryptococcosis was first described by German scientists in a young woman with inflammatory symptoms on the tibia (32). The lungs and brain are the major target organs for the cryptococcus organism causing human cryptococcosis: pulmonary cryptococcosis is caused by the entry of pathogenic airborne spores (conidia) or dried yeast cells into the airway and lungs; Cryptococcal meningitis (CM) is the infection from occasional sits (preferentially from lung or elsewhere such as skin, liver etc.) disseminating during the early phase via circulation into other parts of the body especially to the central nervous system (CNS) (33–35). Cryptococci can utilize parasitized phagocytes (monocytes/macrophages) to cross the BBB into the brain via a Trojan horse smuggling the pathogens in Charlier et al. (36) and Kim (37). The outcome of fungal colonization in the lungs include either clearance of fungi by the host immune system or by establishing an infection from the asymptomatic infection at the latency period, into a virtual local inflammation characterized by pulmonary nodules and pneumonia, or the development of a subsequent dissemination into the systemic organs, preferentially into the CNS through the BBB. Therefore, cryptococci could be detected mainly from lung tissue/bronchoalveolar lavage/sputum or cerebrospinal fluid or blood, and CM has been considered to be severe and life-threatening mycosis with a high mortality derived from cryptococcosis. According to the immune reaction to a diverse polysaccharide capsule, the encapsulated cryptococcus could be classified into four serotypes (from A to D) and two major species are responsible for opportunistic cryptococcosis: the *C. neoformans* (serotype A and D) and *C. gattii* (serotypes B and C), which are infectious in immunocompromised hosts and in immunocompetent or immunologically normal individuals, respectively (32, 38–40). In the AIDS epidemic, CD4^+^ T lymphocytes deficiency renders HIV/AIDS patients less resistant to cryptococci, thus the rise in cases of human cryptococcosis was followed by the increasing diagnosis and morbidity of HIV/AIDS despite the development of antiretroviral therapy (6, 41, 42). The lung serves as a target organ in patients with HIV/AIDS, but once infected, the likelihood of meningitis is high, possibly because the dissemination of cryptococcosis into the brain depends on the status of the pulmonary immune responses (43). On the other hand, highly encapsulated cryptococci are more frequently found in the respiratory tracts rather than the CNS, which resist phagocytes and reduce the rate of systemic dissemination (44). In a basic study, severe combined immunodeficiency (SCID) mice inoculated intravenously (i.v.) with *C. neoformans* viable cells could mimic systemic cryptococcosis in immunodeficient hosts (45). Of note, cryptococcosis in HIV-negative patients is not rare as previously thought (46), these individuals show no apparent immune deficiency but under immunosuppressive conditions including: immunosuppressive drug treatment (glucocorticoids or other immunosuppressants) and malignancies or hematologic disorders (chronic leukemia, lymphoma), the presence of systemic lupus erythematosus patients receiving immunosuppressive agents, and other diseases e.g., diabetes mellitus, cirrhosis, and chronic renal failure, also contribute to cryptococcosis in HIV-negative patients. Interestingly, although the development of the immune system in children is insufficient, pulmonary cryptococcosis in children is rarely observed but can often be a fatal disease (47) (Figure 2A).

Invasive pulmonary aspergillosis (IPA), an opportunistic mycosis, results from a pathogenic Aspergillus infection in the lung. Susceptible individuals on Ap are very similar to those on *C. neoformans, Aspergillus* poses a serious threat to immunocompromised individuals including hosts with cancer or hematological disorders (acute leukemia, neutropenia), patients with chronic granulomatous diseases, and immunosuppressed bone marrow/organ transplant recipients (48–51). In contrast, inhaled infectious propagules in immunocompetent hosts show no further significance because of the strong resistance to the fungus, with killing and clearance by the cells of the pulmonary immune system (Figure 2B).

Regarding the process of human infection establishment, we elucidate that as an “unbalanced fight between host and pathogen,” on one hand, the immunocompromised host or patient with underlying immunosuppressive conditions are characterized by the cellular immune deficiency: impairing the killing and clearance ability of phagocytes e.g., resident alveolar macrophages and weakened cellular immunity mediated by CD4 T lymphocytes, both of which contribute to host resistance to the fungi; in addition, the encapsulated cryptococcus resist the phagocytosis and rapidly germinate into mature yeast cells to establish an infection in the host. For the therapy of mycoses, Amphotericin B (AMB), fluconazole and echinocandins are the optimal antifungal agents (52–54), but occasionally current antifungal drugs may not cure these diseases due to a growing antifungal drug resistance, toxicity and side effects which still pose a potential risks for patients (55). Therefore, novel strategies rather focus on host defense, which is a crucial determinant of fungal pathogenesis. At present, the biggest problem in the treatment of invasive mycoses, is the ineffectiveness of antifungal agents, and new therapeutic immunomodulators are urgently required. Additionally, poor prognosis is attributed to the severe CM in the brain. Therefore, therapeutic strategies for both enhancing the clearance of pathogens and the prevention of dissemination into the CNS, are still required (Figure 2C).

### Pathogen-Host Communication: The Recognition and Internalization of the Fungi by the Host Innate Immune System in Initial Signaling

To understand the communication between host and fungal pathogens would be useful for the diagnosis and therapy of the trajectory of infection. However, the signaling events for fungal pathogens and mammalian host cell interactions remain poorly understood, although some research progress has been made in this direction (56).

The fungal surface is mainly comprised of chitin, modified glycoproteins and glucans. In most yeasts, mannose-containing polysaccharides are often covalently attached to proteins. *C. neoformans* is a unique pathogenic fungus which employs a specific polysaccharide component (glucuronoxylomannan, GXM) as a capsule outside the cell wall, with anti-phagocytosis activity (57). GXM in the *C. neoformans* capsule is essential for virulence of this pathogen. In fact, patients with cryptococcosis accumulate GXM in the cerebrospinal fluid and serum, where it is associated with several immunomodulatory properties.

The fungal surface polysaccharides or glycans are the main PAMPs recognized by host cells, while the surface proteins on different fungi are essential for their virulence and intracellular survival. It is known that phagocytosis by host cells is an early event in the host-microbe interaction (58). The precise mechanisms that mammalian host cells coordinately manipulate fungal phagocytosis and replication with, remain complex and elusive. Professional phagocytes, for instance macrophages, can internalize and kill large particulate material through the phagocytosis process involving reactive oxygen species (ROS) (59). Binding of pathogenic particles to cell surface receptors, with a reorganization of the membrane and intracellular elements, would result in the phagocytosis of particle and phagosome formation. Notably, engulfment of the fungal pathogens triggers the host autophagy initiation complex (AIC) and the upstream kinases, which can mediate autophagy in the *C. neoformans* infection (60).

## Host: Cell Populations in Host Defense Against Pulmonary Fungal Infections

Although mounting signal mechanisms driving the initiation and development of fungal infection investigated by independent groups have been clearly revealed, we have a relatively poor view about the integrated cellular and molecular systems involved in the process of distinct fungal infection in the lung. Thus, effective therapeutic approaches that tackle harmful pulmonary fungal infection and inflammation, remains limited. Host mechanisms of fungal infection in the lung derive from multiple cell types and numerous molecules including receptors, adaptors, kinases, and transcriptional factors in the fungal pathogen challenge. Here we highlight the representative components in the host response, from cell biology and molecule biology focusing on *Aspergillus, Cryptococcus*, and other pulmonary fungi.

**Figure 3:**
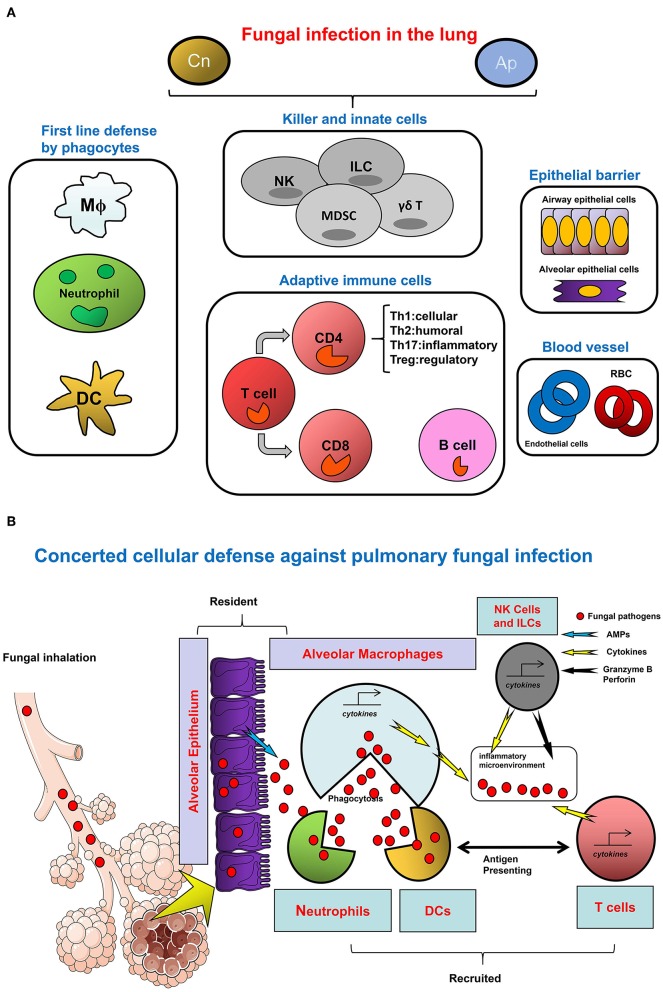
The Network of cellular defense against fungal infections in the lung. **(A)** The cell category in host response to infection. **(B)** Divergent cell types orchestrate the host defense against fungal infection. To defend against pulmonary fungal infection as represented by Cn and Ap, divergent types of cells are involved in the microenvironment of infectious sites in the lung. Among them, granulocytes and monocytes/macrophages as well as DCs are typical myeloid lineages which are the major cellular agents for innate immunity. In the respiratory tract, these cells constitute the first line of defense. Alveolar macrophages are monocytes/macrophages specifically resident in the lung; neutrophils are quickly infiltrated to the sites of inflammation to eradicate the pathogen and to promote tissue repair; pathogens, or antigens bind to DC-SIGN expressing immature DC to gain access to these cells for uptake, meanwhile activating DCs trigger adaptive immunity mediated by the T cells. The direct anti-pathogen effect of NK cells, as a central player of innate immunity, is ascribed to their direct cytotoxicity and cytotoxic cytokine and granule-secreting capacity. In addition, other innate like cells such as ILC and special T cell types such as γδT cells also contribute to the host defense by cytokine secretion or other approaches, with concomitant inducement of MDSC. For adaptive immunity, CD4+ T helper cell immunity is predominant in the pathogen-induced inflammation, IFN-γ produced by active Th1 cells is beneficial for Cryptococcus clearance, and the Th17 cells with IL-17/IL-23 secretion also plays important roles in a pulmonary fungal infection. In contrast, Th2 cells with anti-inflammatory cytokine secretion and Treg cells with immunosuppression activity participate in the host response upon fungal infection usually with negative outcomes. In the microenvironment, secreted cytokines further regulate innate and adaptive cells function through cytokine receptors. Besides multiple immune cells, the epithelial cells at the mucosal barrier also confer a protective activity against the invasive fungi in the respiratory tract. In the lung blood vessel, the roles of endothelial cells and RBC have rarely been reported.

Innate immunity plays a central role in host protection against fungal infection. Invasive pulmonary aspergillosis (IPA), candidiasis, and other mycosis can develop in the hosts with specific immune deficiency defects, such as chemotherapy-induced prolonged neutropenia or functional defects in NADPH oxidase. After the initial sensing of fungi by pattern recognition receptors (PRRs), neutrophils, and macrophages are recruited as first line immune cells to the infected and inflammatory sites for their clearance of fungal pathogens at an early stage of infection. Fungal antigens can be presented by DCs which also bridge innate and adaptive immunity. In fact, the innate immune system orchestrates the first step to protect hosts against fungal infection in the lungs (Figures 3A,B).

### Phagocytic and Myeloid Derived Innate Cells: Alveolar Macrophages and Neutrophils, and Dendritic Cells

Multiple innate immune cells as well as adaptive CD4^+^ Th1 type cells constitute a precise network for the host defense against pulmonary fungal pathogens (61, 62), In particular, lung phagocytic leukocytes including the resident alveolar macrophages (AM), DC and neutrophils often functionally modulate the early immune response to pulmonary infection in the first line with an armored defense against pulmonary fungal infections (63). A study reported that CD11c^+^ AM population is dominant in uninfected lungs as well as in the early days following infection with *C. neoformans*. Thereafter, the rapid expansion of CD11c^+^ cells is attributed to accumulated pulmonary DCs. Here CD11b expression is used to distinguish between AM (CD11c^+^CD11b^min^) and conventional DC (CD11c^+^CD11b^+^). It was indicated that *C. neoformans* infected mice with AM/DC population depletion, displayed very rapid deterioration (63). Alveolar macrophages mount inflammatory responses to fungal pathogens with specific productions of tumor necrosis factor-α, interleukin-1α/β, interleukin-6, macrophage inflammatory proteins, and granulocyte-colony stimulating factor, which mainly depends on signaling from recognized fungal glucan via host PRR dectin-1 (64).

Neutrophil is a key orchestrator during the infectious process, particularly for polymorphonuclear neutrophils (PMN). These cells can initiate quick lung infiltration and arrive at inflammatory sites rapidly where it plays a vital role in eliminating pathogens and promoting tissue repair. At the early phase of *Aspergillus fumigatus* infection, AM and neutrophil coordinately shape the host defense and survival following fungal inhalation, AM is believed to kill conidia while neutrophil is postulated to restrict the tissue invasion of hyphae (61). A well-established method of transient neutrophil depletion by antibody RB6-8C5 (anti-Ly6G/Ly6C) can be used to evaluate the temporal demand for neutrophils in the host defense (65). RB6-8C5 induced neutropenia both in circulation and in single cell lung suspensions without affecting the CD11b+Ly6C+Ly6G– monocytes, leading to invasive aspergillosis and tissue damage (66). Mice who received RB6-8C5 prior to or within 3 h after infection were exacerbated as shown by higher susceptibility and death rates, and this is different from the depletion at later time points when the survival rates are nearly normal. Neutrophil is also a type of IL-17, producing cells in several mouse infectious models. Eric Pearlman demonstrated that in *A. fumigatus* infection Ly6G+Ly6C+ neutrophil is activated through IL-17A-IL-17RC interactions via multiple molecules including dectin-2 and IL-17 signaling related IL-6, IL-23, RORgt (67). In addition, neutrophil activity could be governed by TLRs in Aspergillosis (10). Recognition of *A. fumigatus* conidia trigger non-oxidative intracellular killing involving integrin CD11b/CD18 and PI3K. When the conidia germinate, by escaping from early killing, the extracellular destruction of the *Aspergillus* hyphae requires antibody mediated opsonization with the involvement of Fcγ receptors recognition and kinase signaling for downstream ROS related metabolites production via MPO and NADPH oxidase (68).

Phagocytic leukocytes in the lung such as AM, DC, and recruited neutrophils constitute the first immune defense by phagocytosing pathogens with yeast lysis upon *Aspergillus* or *Cryptococcus* exposure after inhalation. Therefore, the resident AM and DC contribute to the early innate immune response and later adaptive immunity regulation. Dendritic cells (DCs) are the classic innate immune cell type which potentially trigger and control adaptive immunity mainly mediated by T and B lymphocytes (69, 70). Pulmonary DCs also coordinate adaptive immune responses to *A. fumigatus*, by regulating T cell proliferation and enhancing the protective Th1 response within the lung (71–73). Dectin-1 is responsible for immature DCs pro-inflammatory responses after exposure to *A. fumigatus* (74). Interestingly, the neutrophils could mediate lung DC maturation and efflux (75), while DCs trigger the secreted chemokines for neutrophils/Th1 lymphocytes recruitment (76), suggesting the cellular interplays among neutrophils, DCs as well as T lymphocytes.

### NK Cells and T Lymphocytes

Natural killer (NK) cells in the innate immune system confer primary immune protection against tumor and pathogenic microbes through granule-mediated killing and effector IFN-γ release (77, 78). It was found that the adoptive transfer of CD4^+^ T cells are responsible for the increase of NK cells with the activation marker NKG2D and the release of gamma interferon, granzyme B and perforin during *Pneumocystis* infection (79). Moreover, *A. fumigatus* can be recognized by human NK cells with direct response to hyphae but not conidia (80). Unlike other innate immune cells, NK cells do not directly defend the fungus through the phagocytosis process but appear to regulate their antifungal function via the production of inflammatory IFN-γ (81). Similarly, murine NK cells are antifungal to *C. neoformans* by directly killing the organism (82). The precise mechanisms of natural killer cells' mediated defense against respiratory fungal pathogens and their interplay with other immune cells during infection are still limited and require further study.

The homeostasis of multiple adaptive T cell subtypes (typically the CD4 positive subtype including Th1/Th2/Th17/Treg cells) are involved in the further defense against the fungal pathogen after innate immunity. Although the crucial role of the initial innate immunity against pulmonary fungal infection in host-pathogen interplay have been well-described, the functions of adaptive immunity with representative T cell responses are equally of importance for the host's defense. A Th1 type adaptive immune response with Th1 cytokines such as IFN-γ is often required for the clearance of *C. neoformans* yeast. For instance, previous studies have revealed that in mice infected with an IFN-gamma-producing *C. neoformans* strain, H99-gamma were resistant to a second infection in the lung by a lethal strain. In the respiratory tract, increased granulomatous formation, rapid inflammatory infiltration, and Th1 mediated adaptive immunity were involved in protection against cryptococcosis (83). Whereas, the mRNA expression profiles of pulmonary cytokines in infected mice were of Th2-type, IL-12 treatment protected mice from a lethal infection by regulating the host immune balance toward the Th1-state (84). Tregs and Th17 cells with IL-17 production exhibit opposite responses to aspergillosis, excessive inflammation driven by IL-17 augmentation and deduced Tregs with anti-inflammatory activity which led to a higher susceptibility to *A. fumigatus* in mice (85). In addition, CD8^+^ T cell regulation in fungal infection and the molecular mechanisms underlying fungal clearance, remain rarely reported. One study displayed that the protective and antifungal memory of CD8^+^ T cell responses are promoted by TLR3, which could serve as a new therapeutic target for aspergillosis in high-risk patients (86).

### Lung Epithelial Cells

Multiple hematopoietic cells such as macrophages and DCs play crucial roles in anti-fungal immune resistance and tolerance to fungi, by keeping a balance of immunopathology/protective immunity. However, growing evidence implies that the epithelial cells play an essential role in infection and inflammation by releasing surfactant proteins and antimicrobial peptides (87). First, internalization of *A. fumigatus* conidia could also be performed by epithelial and endothelial cells (88). Lung epithelial cells (LEC) often play crucial roles in the crosstalk between fungal infection and mucosal immunity at pulmonary mucosa by linking the innate immunity and adaptive immunity. It is reported that the protective tolerance on LECs to *A. fumigatus* is achieved through indoleamine 2,3-dioxygenase (IDO) signaling (89).

## Key Molecules Regulating Host Immune Responses to Fungal Infections in the Lung

**Figure 4:**
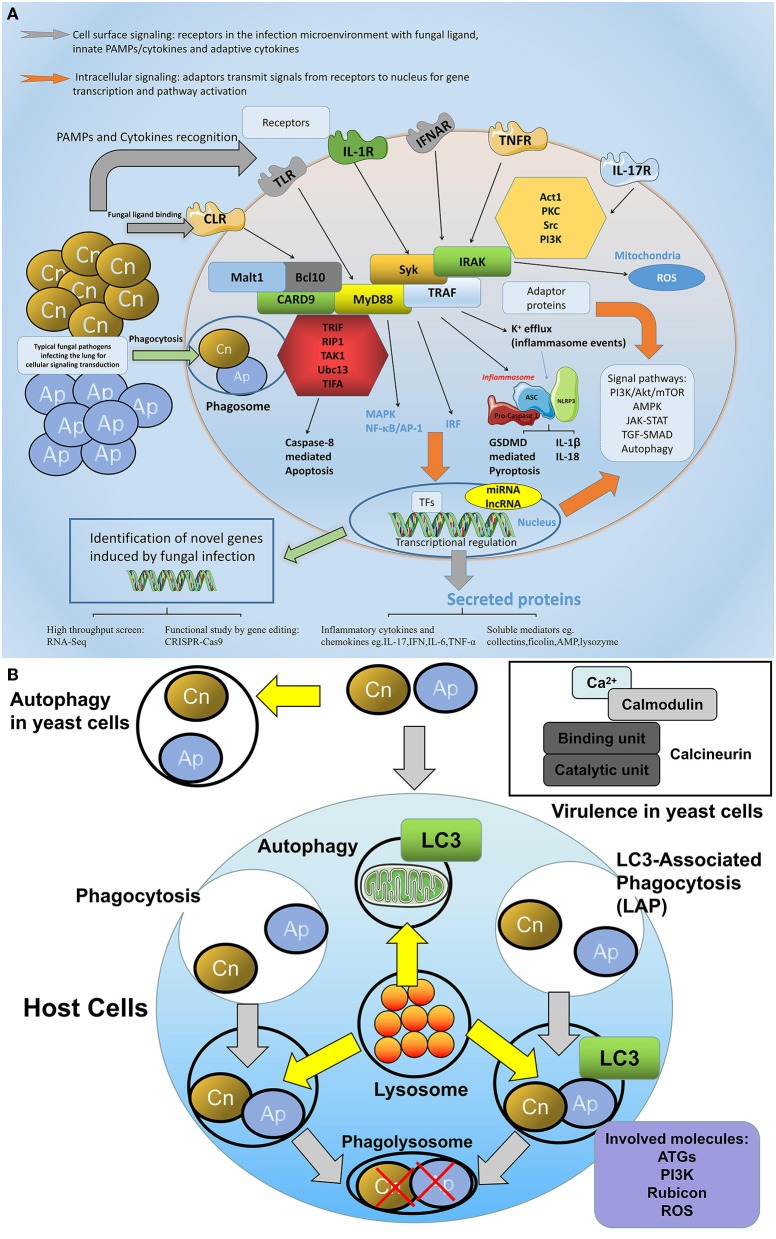
The network of molecular defense against fungal infections in the lung. **(A)** General involved molecules and signal events in the aspects of cellular fungal infection. During fungal infection, various intracellular and extracellular molecules are involved in the host defense process. On the cell surface, receptors especially the PRRs are predominant regulators that sense fungal pathogens or fungal associated PAMPs, among them C-type lectin receptors (CLRs) including dectin-1, dectin-2, and MR play crucial roles in fungi-host communication. Toll-like receptors are also engaged in fungal recognition. In addition, cytokine receptors such as IL-1R, TNFR, or IFNAR are also activated upon receiving the respective cytokine signals. For intracellular signaling, adaptor proteins such as MyD88, CARD9, Syk, and TRAF are able to transmit signals via divergent pathways, leading to MAPK/NF-κB activation or type I interferon production, or inflammasome assembly. These pathways either trigger protein modulation such as translocation, phosphorylation, and ubiquitination, or at a transcriptional level, regulates gene expression in the nucleus, which leads to cell activities such as proliferation, apoptosis, or pyroptosis. **(B)** The autophagy and calcineurin signaling in the host-fungi during infection. The autophagy and calcineurin signaling are important for the host-fungi bioactivity regulation. Autophagy could occur in both yeast cells and host cells. LAP is a special intracellular autophagy which forms to defend extracellular fungi internalized and which combine the characters of both phagocytosis and autophagy: pathogens are entrapped in the phagosome with LC3 recruitment, and the lysosome is involved in the fusion of the phagolysosome where engulfed pathogens are killed by degradation. Key molecules including ATG, PI3K, Rubicon, and ROS play roles in the fungi induced LAP signaling. In yeast cells, calcineurin transmitting calcium and calmodulin signaling is responsible for the virulence of the pathogen.

Various molecules play distinct but crucial roles in innate immunity, the present review also sheds light on molecular regulation, during immune tolerance, to pulmonary fungus (Figure 4A).

### Receptors

#### PRRs Including CLRs and TLRs

Signaling transductions, through host receptors in an infection, are relatively well-studied. In addition to fungi-derived PAMPs (13, 14), some host-encoded molecules, and the damage-associated molecular patterns (DAMPs), are secreted during fungal infection for host defense (90). CLRs and TLRs/IL-1R are classical and predominant PRRs engaged for fungal pathogen recognition. CLRs are a group of important C-type lectin receptors including Dectin-1/2, MCL (Macrophage C-type lectin, Clec4d), Mincle (macrophage inducible C-type lectin, Clec4e), and MR (mannose receptor) that are all involved in fungal recognition (91–94). In a lung infection with *A. fumigatus*, dectin-1 expressed on resident alveolar macrophages and polymorphonuclear neutrophils is required for the initiation of a host response while the role of dectin-2 is not well-investigated (95). Recently, a C-type lectin receptor, and melanin-sensing C-type lectin receptor (MelLec) was identified by the Gordon D. Brown group (96), which was fungicidal by sensing the unit of DHN-melanin in conidia of *A. fumigatus*. In animal models, MelLec was protective in the host against *A. fumigatus*. In humans, a single nucleotide polymorphism of MelLec was found to negatively regulate inflammatory responses by myeloid cells and increased the incidence of *A. fumigatus* infection in stem cell transplant recipients.

*C. neoformans* glycoantigens such as mannoproteins can bind different lectin receptors such as the mannose receptor and DC-specific non-ICAM3 grabbing nonintergrin (DC-SIGN) (97, 98). Phagocytosis of pathogens by myeloid cells upon PRRs recognition or exposure to soluble glycoantigens and cryptococcal DNA, would lead to cytokines/chemokines production as well as yeast lysis. Moreover, various TLRs are distributed on different immune cells, for resident alveolar macrophages, the TLR2, TLR4, and TLR7 are highly expressed and immunosuppressed mice with TLR2 or TLR4 deficiency are more susceptible to invasive aspergillosis (99–102).

#### Cytokine Receptors Such as IL-1R and TNFR

IL-1R shares a high homology with TLRs in signal transduction (103) and TLR/IL-1R signaling is critically involved in the response to fungal infections (104). The complex group of TNF ligands and the receptor superfamily serve as a rich source of drug targets on both innate and adaptive immunity. As a member of the tumor necrosis factor receptor superfamily, the T cell expressed glucocorticoid-induced TNFR-related protein (GITR, TNFRSF18), mediates both natural and acquired immune reactions by its ligand GITRL expressed on APC. Intriguingly, GITR-GITRL could influence TLR2 and TLR4 expression on DC and correlated with T cells response during candidiasis (105). However, the effect of GITRL-GITR on DC and its relationship with Treg during aspergillusis and cryptococcosis remains unclear (106).

#### Adaptor Molecules

The TLR-mediated signaling pathways for downstream NF-κB activation events are important for the host immune regulation against fungal infections. Signaling by the TLR or IL-1R could employ multiple adaptor proteins including MyD88, TRAF6, TRIF, etc. for downstream signal cascades transduction.

#### MyD88

Previous studies on the innate immune mechanisms revealed a remarkable conservation of molecular components of host defense signaling pathways, among them the Toll-IL-1R homology (TIR) domain, presented by adaptor protein MyD88, is essential for signal transduction. The downstream NF-κB activation could be induced by adaptor MyD88 via the human TLR/IL-1R transduction pathway, through IRAK and TRAF6 (107). Under the condition of fungal infection, overexpression of MyD88 was functionally enough for the induction of fungicidal peptide Drosomycin *in vitro*. A recent study found that MyD88 also plays an important role in host defense during a respiratory fungal attack especially at the first phase of infection. Ubiquitous airborne conidia could be formed by *A. fumigatus* and inhaled by humans daily, in this process, adaptors including MyD88 and CARD9 could be activated via host receptors such as the C-type lectin receptor, TLR, and IL-1R for immune signaling transduction as well as fungal clearance. At the onset of respiratory fungal infection, MyD88 depletion delayed neutrophil lung trafficking and chemokines production, leading to injury in the lung. MyD88 expressed on lung epithelial cells, was responsible for rapid neutrophil recruitment and chemokines production through IL-1R. Exogenous CXCL1 treatment also rescued mortality in MyD88-deficient mice. Altogether, host MyD88 associated pathways play essential roles in early responses to *A. fumigatus* in the lung, and fungal PAMPs to host PRRs triggered MyD88-NF-κB signaling, serves as a pivotal pathway for the pathogen-host interaction (100, 108).

#### TRAF Family Proteins

Besides MyD88, TRAF family proteins especially TRAF3 and TRAF6 play a crucial role in regulating innate and adaptive immune responses. In TLR signaling, TRAF6 is dependent on MyD88 instead of TRIF, and TRAF6 differently modulates MyD88- and IRAK-1-induced activation of NF-κB (109, 110). Although TRAF6 and TAK1 are implicated in candida infection (111), the roles of these molecules in CLRs initiated signaling have not been studied. In canonical CLR signaling, both TRAF6-TAK1 interaction and spleen tyrosine kinase (Syk) phosphorylation are initial events involved for CARD9-Bcl10-MALT1 complex and downstream MAPK and NF-κB activation, adaptor Syk-coupled CLRs including Dectin1/2 and Mincle mediate innate immunity against fungal infection (92, 93, 112). Another study proved that Dectin-1-Syk and autophagy contributed to maturation of the *A. fumigatus* phagosome (113).

### The Inflammasome Complex

#### Inflammasomes

The inflammasomes comprised of several intracellular proteins are functionally essential for the regulation of innate immune responses. These complexes could be activated through PAMPs-PRRs or host derived DAMPs signaling and are intensely associated with various infectious pathogens including fungi, viruses, and bacteria especially in a respiratory tract infection (114, 115).

#### NLRP3 and AIM2

NLRP3 and AIM2 are typical inflammasomes which play critical roles, with distinct mechanisms, in innate immunity: The NLRP3 inflammasome activation is regulated by two major steps of signals. First is the priming step in which NLRP3 and pro-IL-1β expressions are induced in a NF-κB dependent manner by PAMPs e.g., TLR agonists (LPS as representative one) or key cytokines e.g., TNF-α and IL-1β. The second step is NLRP3 assembly activation by multiple stimuli e.g., ATP/nigericin. Further studies showed that phagocytosis of particulate matter triggers K^+^ Efflux and activates NLRP3 (116, 117), but recently a K^+^ independent activation mode was described via targeting mitochondria using small molecules (118). As to the biological function of the NLRP3 inflammasome in the field of infection, it plays a crucial protective role against several pulmonary fungi which could cause aspergillosis and cryptococcosis (90). Notably, NLRP3 inflammasome mediated signaling in the host immune defense is recognized by fungal polysaccharides such as curdlan (119, 120), and specifically *A. fumigatus* triggers the activation of the NLRP3 inflammasome via syk kinase activity and reactive oxygen species (121). In addition, fungal zymosan and mannan stimulated the NLRP3 inflammasome to induce macrophage and DC caspase-1 activity as well as IL-1β secretion, suggesting that conserved cell wall components are responsible for the ASC and NLRP3 inflammasome activation during fungal infection (122). In cellular and animal models, acapsular mutant strain of *C. neoformans* (Cap59) but not the encapsulated wild type strain (H99), activates the inflammasome (123). Moreover, the germline NLRP3 knock-out mice infected with biofilm of clinical *C. neoformans* strain HS1101 displayed a more severe infection and inflammation in the lung, which is also true for Casp1 KO and ASC KO mice. These data illustrate the importance of the NLRP3 inflammasome components in the host responses to the fungal challenge in the lung (124). Absence in melanoma 2 (AIM2) is a unique DNA-sensing receptor. Mice lacking AIM2 alone displayed similar susceptibility compared with wild type mice upon *Aspergillus* infection, meanwhile mice with double knockout of AIM2 and NLRP3 failed to control *Aspergillus* hyphae dissemination, thus they succumbed to the attack of fungus more rapidly than wild type mice or mice deficient in either AIM2 or NLRP3 (125). Until now, the functions and mechanisms of other novel host inflammasomes such as NLRP1, NLRC6, NLRP7 in controlling fungal infection, remain elusive.

#### NF-κB and MAPK

NF-κB plays a critical role in the control of infection as well as inflammation, it was shown that the macrophage dectin-1, TLR2 and TLR4 could specifically recognize the mature hyphal forms of *Aspergillus* but not spores, resulting in NF-κB dependent inflammatory cytokine secretion and antimicrobial ROS production (126). As to dectin-2, it was also reported that IκBα (inhibitor of the kappa B protein) phosphorylation and NF-κB activation following *A. fumigatus* stimulation occurs through dectin-2-Syk signaling (127). Interestingly, gliotoxin as a toxic metabolite synthesized by *A. fumigatus* specifically inhibits NF-κB activation and induces apoptosis (128). However, another paper found that ERK but not p38 was essential in the MAPK pathway responsible for defense against *A. fumigatus* on Alveolar Macrophages, whereas NF-κB activation appeared to play a secondary role. After phosphorylation by upstream molecules, the MAPKs translocated to the nucleus to phosphorylate downstream target molecules which transcriptionally regulated cytokine genes (129). Different from macrophage detecting *Aspergillus* via TLR2/4 to trigger TLR-MyD88-NF-κB-dependent synthesis of inflammation related molecules, pulmonary epithelial cells could sense sprouting but not resting spores of *A. fumigatus* to induce the synthesis of interleukin (IL)-8 through p38 MAPK and ERK1/2 and PI3 kinase, suggesting that MAPK is of importance both on phagocytes and epithelial cells (130). In addition, TLR4 in conjunction with CD14 could be involved in the host defense against *C. neoformans* capsular polysaccharide glucuronoxylomannan (GXM), stimulating NF-κB nuclear translocation in macrophages without MAPK activation and TNF-α release (131).

#### Cytokines and Chemokines

Cytokines/chemokines are predominant modulators secreted from the host in response to various fungal pathogen infections. An imbalance between pro- and anti-inflammatory cytokines might negatively result in infectious diseases due to an impaired host defense.

#### IL-1 Family Cytokines: IL-1β/IL-18/IL-33/IL-36

Until now, 11 members were identified in the IL-1 family of cytokines with seven proinflammatory cytokines including IL-1α, IL-1β, IL-18, IL-33, IL-36α, IL-36β, and IL-36γ and four with anti-inflammatory cytokines including IL-1Ra, IL-36Ra, IL-37, and IL-38 (132). IL-1α and IL-1β are thought to be the primary drivers of inflammation in chronic granulomatous disease via decreased autophagy and increased inflammasome activation (133). With respect to immunopathogenic effects of IL-1 family members during invasive pulmonary aspergillusis (IPA), both lung homogenates and alveolar macrophages derived from Dectin-1 KO mice demonstrated reduced IL-1α/IL-1β and TNF, MIP, and KC *in vitro* (134), suggesting that IL-1α and IL-1β production is derived from Dectin-1 signaling. A recent study has indicated that IL-1R was critical for protection, as IL-1α was required for lymphocytes recruitment and IL-1β conferred resistance to fungal dissemination (135). Members of the IL-1 family often play protective roles in immune defense against the opportunistic mold *A. fumigatus*. Besides IL-1β, IL-18 is also protective against *C. neoformans* by inducing IFN-γ. IL-1β/IL-18 is primarily secreted in a NLRP3 inflammasome-dependent manner as mentioned above. In a recent study, the IL-1 family member IL-33 played regulatory roles in lung infection defense against *A. fumigatus*. IL-33 expression was detected in the lung and increased after exposure to the fungus, independent of Dectin-1. Mice lacking the receptor for IL-33 (Il1rl1^−/−^) unexpectedly demonstrated enhanced lung clearance of the fungal pathogen while IL-33 administration to normal mice inhibited fungal-induced IL-17A and IL-22 through PGE2. Because normal mice produced less PGE2 after fungal exposure when administered IL-33, and PGE2 was significantly increased in fungal-exposed Il1rl1^−/−^ mice, suggesting that IL-33-mediated regulation of IL-17A and IL-22 occurred at the level of PGE2 This was confirmed by cyclooxygenase 2 or PGE2 inhibition, which attenuated fungal-induced protective IL-17A and IL-22, as well as IL-1α, IL-1β, and IL-6 productions in Il1rl1^−/−^ mice, resulting in impaired fungal clearance (132). Moreover, interleukin-36γ (IL-36γ) is a newly identified IL-1 family inflammatory cytokine which is highly expressed on epithelium and some myeloid cells. Studies in humans have shown that *A. fumigatus* strongly induces IL-36g and IL-36Ra, but not IL-36α, which were dependent on dectin-1 and TLR4 while Inhibiting IL-36 signaling was found to abrogate the induction of protective Th17 and Th1 responses (136).

#### IL-17 and IL-23

IL-17 (IL-17A) is a pleiotropic cytokine which has been implicated in the pathogenic development of autoimmune inflammations such as rheumatoid arthritis (RA) and multiple sclerosis (MS), but this cytokine has also been associated with protection against bacterial infections (137). IL-17 could be synthesized more rapidly by natural killer T cells (NKT cells), γδ T cells and innate lymphoid cells than Th17 cells due to RORγt expressed on these cell types (138). The roles of IL-17 and IL-23 in the host response against the pulmonary fungal infection have been of focus recently. In the airway lesions, IL-17 potently regulates neutrophil recruitment and homeostasis together with IL-23 (139, 140). Intriguingly, in the *A. fumigatus* model of pulmonary fungal infection via the intranasal route, IL-17 and IL-23 are highlighted with a regulatory role, by restricting the IL-12-IFN-γ mediated Th1 protective response or even by impairing the antifungal immune resistance (141). Thus, the multi-faceted roles of host inflammation are that it is not only critically responsible for the antifungal immunity, but that is also negatively controls proper immune reactions or even worsens fungal diseases when the balance between protection and pathogenesis is dysregulated in certain milieu.

#### Type I and Type II Interferons

IFN-α/β, as typical type I interferons, are produced by various cell types after many stimuli, especially viruses. Through binding to a specific IFN-α/β receptor termed IFNAR (IFN-α/βR), downstream IFN-stimulated genes (ISGs) can be induced. IFNAR is composed of two subtypes IFNAR1 and IFNAR2. Canonical type I IFN signaling triggers the Janus kinase (JAK)-signal transducer and activator of the transcription (STAT) pathway, resulting in ISG gene expression (142). Up to now, limited information is known about their roles in non-viral infections such as a pulmonary fungal infection (143). Nonetheless, signaling of type I interferons in the host defense against *C. neoformans* was addressed in a recent study. Using IFNAR1 defected mice (IFNAR1 KO), the authors found an increased fungal clearance by, with enhanced Th2 and Th17 responses after infection compared with control animals. In addition, IFNAR1 KO mice exhibited significantly higher MUC5AC expression in bronchoepithelial cells. Therefore, type I interferons might negatively control the early host defense to a fungal infection (144). In contrast, another group reported that mice lacking either the IFNAR or IFN-β succumbed to unrestrained pneumonia and encephalitis after an intratracheal or i.v. challenge with *C. neoformans* with increased Th2 cytokines IL-4/IL-10/IL-13, but reduced TNF-α, IFN-γ, iNOS, and CXCL10, suggesting that the protection by the type I IFN signaling is associated with type I cytokine polarization (145). In the case of *Aspergillus* infection, its conidia mainly induce the IFN-β signaling in respiratory epithelial cells. Resting conidia can be sensed by differentiated human bronchial epithelial cells (HBECs), leading to the production of IFN-β-inducible genes such as IP-10 (CXCL10). Activated T cells could be further regulated by IP-10 (146). Inhibition on the IFN-β signaling mediators such as RIP-1 (Receptor-interacting protein 1) and TBK-1 (TANK-binding kinase-1) by resveratrol could reduce the IFN-β/IP-10 expression (147). On the other hand, Th1 type cytokine IFN-γ has been proven effective in clearing *C. neoformans* infection. Recombinant IFN-γ administration reduces the fungal burden and increases survival rates, reinforcing the efficiency of fungicidal amphotericin B (148, 149). Pulmonary cytokine analysis demonstrated that there was a Th1-type pro-inflammatory cytokines bias, rather than Th2-type cytokines expression, in mice challenged with the IFN-γ releasing *C. neoformans* strain, compared to wild-type strain-treated mice (150).

#### Classical Cytokines and Chemokines: IL-6, IL-8 (KC), TNF-α, GM-CSF

Pro-inflammatory cytokines such as IL-6, IL-8 (KC), TNF-α, and GM-CSF are essential factors in innate immunity. In hematological patients combined with IPA, levels of interleukin (IL)-6 and IL-8 are elevated in both serum and bronchoalveolar lavage fluid (BALF) (151). Circulating IL-6 is able to induce acute phase responses to control local or systemic acute inflammation with its anti-inflammatory activity (152). IL-6 increases *Aspergillus*-induced IL-17 production from healthy and hematological controls, but not in IPA patients with impaired T cell responsiveness to IL-6 (153). The interleukin (IL)-8 as CXC chemokine (CXCL8) is produced from respiratory epithelial cells via the PI3K and MAPK pathways, instead of the TLR-MyD88 pathway, upon *Aspergillus* infection (130), similar to other CXC chemokines such as KC (keratinocyte-derived chemokine, CXCL1, specific for mice, it is the counter chemokine for IL-8 in humans) and MIP-2 (macrophage inflammatory protein 2, CXCL2/3). Markedly higher levels of KC and MIP-2 were observed in the lungs of animals transiently depleted of neutrophils upon challenge with AF; and the lung-specific overexpression of KC improved the outcome of mice in IPA by augmenting the host defense against *Aspergillus* (154, 155). Transcriptionally, proteases derived from *A. fumigatus* elicit cytokine production in human alveolar type II epithelium-like cells by enhancing IL-6 and IL-8 mRNA levels (156). However, some other study showed that limited levels of these cytokines (IL-6 and IL-8) are synthesized by A549 cells after contacting *A. fumigatus*, resulting in impaired recruitment of leukocytes to the lesion sites and pathogen escape from immune defense (157). There is also a study showing that *A. fumigatus* induced an acute inflammation regulated by neutrophils with pro-inflammatory cytokines (TNF-α, GM-CSF, and IL-1β) and chemokines (MIP-1a, MCP-1 and MIP-2) induction during the peak of infection in the lung, neutralizing TNF-α or GM-CSF decreased neutrophil influx and delayed fungal clearance (158). As for TNF-α, it enhances host responses to *A. fumigatus*, and inhibition on its function might increase susceptibility to aspergillosis (159). In a cytokine network assay in the lungs of immunocompetent mice, endogenous TNF-α, IFN-γ, IL-18, IL-12 are immunoreactive with antifungal activity in response to inhaled *A. fumigatus* (160). In addition, Granulocyte-macrophage colony-stimulating factor (GM-CSF) is a pleiotropic hematopoietic cytokine regulating the myeloid cell host response. The receptor of GM-CSF (GM-CSFR/Csf2r) is composed of GM-CSFRα/Csf2ra and GM-CSFRβ/Csf2rb (161). Mice lacking the GM-CSF receptor β chain (GM-CSFRβ) were susceptible with a higher mortality to *A. fumigatus* conidia infection in the lung, illustrating the functional role of GM-CSFRβ signaling against inhaled *A. fumigatus* (162). In response to *C. neoformans* yeast cells or the major capsular polysaccharide-glucuronoxylomannan (GXM), human polymorphonuclear leukocytes from normal subjects can release inflammatory cytokines (163). Of note, macrophage polarization status is critical for the control of *C. neoformans* infection, and IFN-γ and IL-4 are responsible for the M1/2 polarization associated fungicidal activity. This implies the communication of cell mediated immunity and cytokine mediated immunity (164). Considering clinical applications and translational medicine, targeting the cytokines might be a useful intervention approach for antifungal therapy. To this end, a fluoroquinolone drug moxifloxacin conferred protective effect on human monocytes infected with *A. fumigatus* by inhibiting inflammatory cytokine productions via NF-κB and MAPK inactivation (165).

#### Secreted Soluble Proteins in the Lung

Besides the cytokine/chemokines produced by infected immune cells, the innate immune system can form another barrier on the airway surface upon inhalation of fungal pathogens. Numerous soluble peptides and proteins in this barrier confer antimicrobial activity. This is fulfilled by lysozyme, lactoferrin, secretory leukocyte proteases, secretory phospholipase A2, defensins, and cathelicidins, which are largely secreted by cells in the airway submucosal glands or epithelial cells. Just like antimicrobial peptides (AMPs) in the gut, these soluble effector molecules have been shown to eliminate a spectrum of microorganisms with neutralizing, opsonization, antibiotic or direct killing activities (166, 167).

#### Collectin

The human collectin family (collagen-like or C-type lectin) include MBL (Mannan binding lectin) and pulmonary surfactant proteins A to D, which are important innate immune mediators for antifungal defense (168). The functions of collectins encompass opsonization, inflammation regulation, and the direct clearance of pathogens (169). A previous study has demonstrated the protective effect of MBL in a mouse model of aspergillosis (170). In the airways, surfactant proteins render immune protection against fungal accumulation and then clearance by phagocytosis (171). Among them, SP-A in respiratory tract binds to *C. neoformans* without enhancing phagocytosis, while SP-D may play an essential role at the early stage of infection with an increased rate of uptake and phagocytosis. Thus, a greater number of phagocytosed *C. neoformans* cells in wild-type mice than in SP-D KO mice were observed. However, SP-D enhances fungal survival in macrophages *in vitro*, and mice lacking SP-D are protected *in vivo* (171, 172). The reason for these opposing findings awaits further investigation. Collectins, ficolins, and pentraxins are circulating proteins which could serve as opsonins (173). Type II alveolar epithelial cells could secrete H-Ficolin as innate immune opsonin to involve in pulmonary defenses against fungal infection. Silke Schelenz1s' group found that H-ficolin was involved in *A. fumigatus* defense through the activation of the lectin complement pathway, highlighting the interaction between host and fungus and the modulation of the immune response by ficolin (174). In addition, another human serum opsonin, L-ficolin was detected in the bronchoalveolar lavage (BAL) fluid from patients with a fungal infection. L-ficolin opsonization increased IL-8 production from A549 cells and enhanced conidial uptake and the killing of *A. fumigatus* by macrophages and neutrophils, with a reduced release of inflammatory cytokines (175).

#### Defensin and Lysozyme

The release of inflammatory mediators such as AMPs from epithelial cells is a critical step for the generation of protective activities, including inflammatory cell recruitment and generation of direct antimicrobial factors. Defensins, especially the b-defensin, are one type of AMP that are characterized by the presence of b-sheets stabilized by two disulfide bonds. In a study on HBECs, exposure to *A. fumigatus* led to the expression of b-defensin2 and b-defensin9 (hBD2 and hBD9) genes, suggesting that AMPs from respiratory epithelium are involved in host response during *Aspergillus* infection (176). In addition, as one of the most abundant antimicrobial proteins in the airway, lysozyme is a small enzyme synthesized by epithelium as well as resident macrophages in human lung tissues, which contributes to hyphal disruption of *A. fumigatus* (177, 178). In mice, two isoforms of lysozyme are identified: lysozyme P is expressed on Paneth cells in the small intestine and lysozyme M is predominantly expressed on alveolar macrophages, alveolar type II epithelial cells, and bronchoalveolar lavage fluid (BALF) in the lung (179, 180). However, whether lysozyme M renders protection against pathogenic fungi in the lung remains uncertain. The results of previous studies indicate that defensins and lysozymes might be involved in host defense against respiratory fungal infections.

#### Calcineurin Signaling

Calcineurin and related pathways are implicated in the controls of hyphal synthesis, morphological character, and virulence in *A. fumigatus*. Calcineurin is a heterodimer consisting of catalytic subunit A and Ca^2+^/calmodulin binding unit, a mutant of *A. fumigatus* with depleted calcineurin A catalytic subunit displayed deficiency in morphology and decreased filamentation (181–183). Meanwhile, calcineurin mutant with decreased beta-glucan amounts promoted the fungicidal activity of cell wall inhibitors, implying that targeting calcineurin can be a potential synergistic therapy with other fungicides against *A. fumigatus* (184). The growth of *C. neoformans* was sensitive to CsA and FK506 which mediates the signal transduction on Ca^2+^-regulated protein phosphatase calcineurin and the Calcineurin mutant strains failed to be infectious in an animal model of cryptococcosis in the brain, suggesting that calcineurin is necessary for pathogenicity of *C. neoformans* (185) (Figure 4B). In addition, host calcineurin signaling is also important for host immune responses to fungal infection, for instance, the intervention of neutrophils ability on *Aspergillus* species germination was inhibited in hematopoietic stem cell transplant (HSCT) individuals and this impairment was partly attributed to the administration of calcineurin inhibitors (186), suggesting that calcineurin signaling might affect neutrophils activity against *Aspergillus*, especially in immunocompromised hosts. Besides the neutrophils, calcineurin signaling has also been associated with macrophages. It was reported that calcineurin inhibitor tacrolimus impaired macrophage immune responses and the clearance of the major mold pathogen *A. fumigatus* from the airway, possibly due to the inhibition on *A. fumigatus*-induced phagosomal TLR9-BTK-Calcineurin-NFAT cascades independent of MyD88, and NFAT collaborating with NF-κB contributed to TNF-α production in primary alveolar macrophages (187). By targeting host and pathogen calcineurin signaling, cyclosporine (CsA) can be used as both an immunosuppressive drug and antimicrobial agent, which inhibits the protein phosphatase calcineurin. However, inhibition on host calcineurin or its downstream calcineurin-NFAT pathway by CsA, can protect transplant recipients from severe transplant rejection, and it might at the same time increase host immune defects leading to organ transplant-related invasive aspergillosis. Interestingly, the diversity of immunosuppressants determined different outcomes in transplant patients but the death rate appeared to be lower in individuals who received the calcineurin-inhibitors (188), the precise mechanisms on the host calcineurin signaling in cryptococcosis remains to be investigated.

#### Molecules Mediating Autophagy During Fungal Infection

Fungi often infect mammalian hosts where they interplay with each other: extracellular pathogens could be cleared by immune cells via a phagocytosis process, however, pathogens could also induce autophagy which is a lysosomal degradation process essential for host cell survival and homeostasis (189–192). The autophagy machinery may functionally protect against the threats of infection by eliminating invading pathogens (193, 194), specifically, LC3-associated phagocytosis (LAP) in phagocytes is a non-canonical autophagy pattern, and phagosomes with a single-membrane formation followed by a clearance of engulfed pathogens is required in this process. This LAP is started by the sensing of pathogens via PRRs and the LC3 recruitment to the phagosomal membrane. This precise xenophagy or LAP is utilized for the host defense against invading fungi (195–197).

In both host cells and in yeast cells, the extent of autophagy is critical for the control of pathogens as well as the survival of immune cells, mainly because autophagy is associated with cell programmed necrosis and apoptosis (191). In a reported study, the autophagy in macrophages with response to *C. neoformans* was assessed and the non-activated bone marrow derived macrophages (BMDMs) from Atg5 (Autophagy-related gene 5) knockout mice restricted *C. neoformans* growth with fungistatic activity; and *in vivo*, mice with myeloid depletion of Atg5 consistently displayed reduced susceptibility to *C. neoformans* (198). LAP is essential for host anti-fungal responses. Recent evidence indicated that LAP could be blocked by *A. fumigatus* cell wall melanin for its pathogenicity enhancement (199, 200). Canonical autophagy molecules are equally of importance for LAP, for instance, beclin-1 in the PI3K pathway is recruited to autophagosome after internalization of zymosan by phagocytes. The autophagy protein ATG7 is also important for LAP, because the loss of this molecule would lead to the abolishment of LC3 recruitment and reduced clearance of internalized pathogens and apoptotic cells (201, 202). The recently identified autophagy protein Rubicon (RUN domain protein as Beclin-1 interacting and cysteine rich containing) was essential for LAP but not autophagy induction, mediating clearance of A fumit (203). In addition, NADPH regulated ROS is required for LC3 recruitment (200).

Interestingly, the Atg genes in *C. neoformans* are also involved in the pathogenic mechanisms of infection, for instance, *C. neoformans* with an Atg7 mutant conferred lower survival but higher susceptibility to the killing machinery of different host phagocytes in mice with a pulmonary infection (204). It was proven that PI3K signaling with a defective formation of Atg8 labeled vesicles within the *C. neoformans* exhibited a dramatic virulence attenuation in a murine infection model (205) (Figure 4B).

## Perspective

In this review, we summarized the cellular and molecular mechanisms of the host defense against pathogenic fungal infections in the lung. Regarding cells, innate immune cells including macrophages, neutrophils as well as DCs are the first line cells in the host response to a fungal invasion; adaptive T lymphocytes are of importance to restrain fungal expansion; and the NK cells are responsible for the direct killing of fungal pathogens by its cytotoxicity. In regards to signaling molecules, receptors-adaptors-transcriptional factors form the cascade to maintain homeostasis upon fungal infection. Cytokine production is essential in the microenvironment as they can link intercellular communication via cytokine receptors. However, more mechanisms remain to be studied. Phagocytosis regulation, autophagy, miRNA/lncRNA or ROS signaling in pathogenic fungal infections are all currently understudied. Moreover, the identification of novel genes and pathways via high throughput screening or sequencing and further functional studies are still required to elucidate the host defense mechanisms against infectious fungal pathogens in the lung.

## Author Contributions

ZL and GM conceived, wrote, and edited the manuscript. GL and GM checked and approved the final version of the manuscript.

### Conflict of Interest Statement

The authors declare that the research was conducted in the absence of any commercial or financial relationships that could be construed as a potential conflict of interest.
